# Perceptions of Barriers: An Examination of Public Health Practice in Kansas

**DOI:** 10.3390/ijerph19095513

**Published:** 2022-05-01

**Authors:** Megan Eppler, Kayla Brock, Cheyenne Brunkow, Ellyn R. Mulcahy

**Affiliations:** 1 Master of Public Health Program, College of Veterinary Medicine, Kansas State University, Manhattan, KS 66502, USA; megeppler96@ksu.edu (M.E.); kbrock@ksu.edu (K.B.); brunkowchey@ksu.edu (C.B.); 2 Department of Diagnostic Medicine and Pathobiology, College of Veterinary Medicine, Kansas State University, Manhattan, KS 66502, USA

**Keywords:** public health, public health practitioners, barriers, Kansas

## Abstract

Public health and healthcare professionals perform a wide variety of services for their communities, and serve in important and often overlapping roles, particularly in rural communities. In this qualitative study, public health practitioners in Kansas were asked about their perceptions of barriers to public health and vulnerable people in their communities. Participants from across Kansas were interviewed via teleconferencing, telephone, or email, and emergent themes were identified using qualitative thematic analysis. While asked about public health specifically, during interviews, many participants discussed barriers to healthcare as well. The top barriers to effective public health practice identified in this study were funding, education, accessibility, and affordability. Others included politics, transportation, and the need to expand Medicare and Medicaid. The populations believed most vulnerable in their communities were community members living in poverty, elderly people, and other marginalized populations. Our findings suggest public health practitioners in Kansas observe a lack of understanding and knowledge in their communities about public health, along with the recognition that a lack of accessibility and affordability to health services are barriers to effective public health practice.

## 1. Introduction

Public health, as a structural mechanism to protect population health, addresses and prevents inequality and inequity in communities [1]. However, vulnerable populations may not benefit from equal opportunity or fair access to healthcare services as is intended by equitable health or public health infrastructures [2]. At global and national levels, surveillance and analysis of disparities are vital for tackling social determinants of health that impact our public health [3]. At the state level, it is also important to assess how public health practitioners perceive barriers to local public health. This is particularly important in states with large rural areas where health departments also provide direct healthcare services, impacting their ability to provide population health programming [4,5,6,7].

When studying Kansas public health, it is important to consider its demographics, in addition to healthcare and public health landscapes. Kansas has 105 counties, with 87 that are entirely rural [8,9]. For this large rural expanse, there are 29 short-term hospitals, 42 federally qualified health centers, 82 critical access hospitals, and 180 rural clinics [10]. In addition to a complex healthcare pattern, public health is decentralized, with no singular model. One local health department (LHD) is run by a federally qualified health center, two LHDs are multi-county serving seven counties, two LHDs are city-county, four LHDs are run by county emergency medical services agencies, and four LHDs are run by hospitals [8,11,12]. This totals 18 counties served by non-traditional departments, and 97 county-operated LHDs for each of the remaining counties, [8,11,12]. Therefore, the healthcare and public health model in Kansas is a patchwork of access points for different communities.

Measures of social determinants of health in rural versus urban areas in Kansas are stark, with several indicators worse for rural counties (see Table 1). In 2019, the rural poverty rate was 12.9%, compared to 10.6% in urban Kansas and 12% at the state level [10,13,14]. The median rural family income was USD 65,100, compared to USD 85,200 in urban Kansas and USD 77,400 at the state level [13,14]. A total of 27.5% of rural adults smoked, compared to 17.4% in urban counties and 16.2% at the state level [15,16]. The exercise opportunities were 59.5% in rural counties, compared to 80.1% in urban counties and 80.4% in Kansas as a whole [17,18]. Lastly, rural Kansas reported 10.6% of their population as lacking health insurance, compared to 9.8% of urban, and 9.2% at the state level [19,20,21] (see Table 1).

Public health practice is described at the state and national levels in the US, and local public health may not always be included in these analyses [22,23,24,25,26]. Kansas public health practice has not been extensively studied outside analyses of public health workforce, community health assessment, and public health training needs [27,28,29,30,31]. In addition, many LHDs in Kansas are considered small in size and therefore are not often included in national surveys of LHDs [8,11,12,25]. To address this knowledge gap of local public health practice, it is important to determine what barriers and challenges public health practitioners face in a decentralized and complex public health system, as found in Kansas.

The main aim of this study was to conduct a qualitative thematic analysis of interviews with public health practitioners, regarding perceptions of barriers to public health and vulnerable people in their communities. Kansas public health practitioners identified several barriers to public health and marginalized populations, suggesting an important lack of public health resources in their communities, and confirming that concerns regarding public health nationally in the US are reflected in local public health in Kansas.

## 2. Materials and Methods

The study participants were recruited from all six public health regions of Kansas as utilized by the Kansas Department of Health and the Environment (KDHE) [32]. All potential participants were contacted twice by email and telephone. The potential participants were identified as city or county employees with public health employment titles identified from publicly available information, including community health, emergency services, environmental health, public health, animal health, transportation, aging, and health education. Of a total of 314 potential participants identified in six regions, the final number of participants recruited met the following criteria: they self-identified as public health practitioners, agreed to participate, and completed the informed consent. Interviews took place over email, phone, or Zoom. These participants were located in 14 counties in five of the six public health regions: Northeast, Southeast, East Central, Central, and East [32]. The participants were located in counties with LHDs identified as federally qualified health centers, city-county LHDs, county emergency medical services agency LHDs, or traditional single-county LHDs [6,9,10]. The number of participants (25) for qualitative thematic analysis was determined to be sufficient via utilization of code meaning as a method of thematic saturation. This ensured that the appropriate number of participants were interviewed to arrive at a certainty that all codes were identified in the dataset [33,34,35].

Interviews were transcribed immediately after they were conducted to ensure accuracy. To achieve thematic saturation, the authors reviewed the transcripts for meaning until they reached a full and clear understanding of themes [33,34,35]. A theme or code in qualitative analysis is a word or phrase that summarizes or captures the essence of a portion of data. The transcripts were reviewed, and each theme (or code) was identified. In each interview transcript, new themes (or codes) were identified, until no other new theme emerged. At this point, the authors established that the code has reached saturation. No more participants were recruited into the study, and no further interviews were conducted.

The transcripts and notes were read again independently by all authors. Codes were assigned for qualitative analysis of thematic content, with themes related to public health, barriers to public health, and vulnerable populations. To ensure accurate coding, and consistent and reliable identification of themes, the authors discussed and agreed on the identified recurring patterns and emerging themes. This method ensures the process of coding is systematic and the coded data collection is consistent. The corrected, typed transcripts, and notes were entered into NVivo12 Plus software (QRS International Ltd., Burlington, MA, USA, 2018) to sort and analyze the data [36,37].

The final study population was made up of 25 practitioners from 14 counties in the State of Kansas who self-identified as public health practitioners. The study protocol was approved by the institutional review board (IRB#: 10187, 10187.1) of Kansas State University and informed consent was obtained for all participants.

## 3. Results

Twenty-five public health practitioners from across the state of Kansas were interviewed on their perceptions of barriers to public health in their communities. Of the total study population, 96% self-identified as white, 52% as female, and 48% as male. The ages of participants ranged from 20 to 70 years, and the level of educational attainment ranged from a secondary school level diploma to a doctoral degree. The barriers identified and vulnerable populations recognized in this study are described below, and key findings are reported in Table 2 and Table 3.

### 3.1. Barriers to Public Health

#### 3.1.1. Funding

Funding was the most common barrier to public health, identified by 17 participants (See Table 2). When asked what the biggest barrier to public health was, one participant said, “Funding and politics”. Participants remarked that it is challenging to implement health policies with a lack of funding and were aware of health disparities and inequities in their communities. Some public health practitioners interviewed stated that they would be willing to provide educational outreach and recognized their limitations with little or no funding.

“*Programs receive no funding at all, or we have to share funding with other programs*”

“*We are unable to alleviate the problems in our communities due to funding issues*”

#### 3.1.2. Education and Knowledge

A lack of education or knowledge was the second most common barrier to public health, identified by 15 participants (See Table 2). Participants recognized that community health depends on its citizens to know their health risks. Participants noted that even though people may be aware of their individual health risks, they may not be aware of population-based public health programs in their community. Many participants noted that comprehensive health education programs in schools can help children lead a healthy lifestyle into adulthood.

“*Community education on available public health programs can help improve the community’s health by exposing them to services available*”

“*Even if health programs can tackle the most significant obstacle [funding] we face community awareness challenges*”

#### 3.1.3. Accessibility and Affordability

Fifteen participants noted that health insurance and the cost of healthcare services is expensive, especially for low-income citizens (See Table 2). Specifically, participants discussed lack of affordable health insurance, access to affordable care, and lack of equity in availability of services.

“*Private practices only accept a certain number of patients who pay with monthly payments*”

“*People cannot afford or do not want to go into debt for health services, so they are less likely to seek treatment unless necessary...symptoms may progress much further than if treated in the early stages*”

#### 3.1.4. Politics

Politics was identified as a barrier to public health. Participants observed public health becoming a political issue and had experienced mistrust in public health officials and medical professionals (See Table 2). Ultimately, participants recognized that policymakers determine funding for public health programs, and therefore, policymakers need to be aware of the needs of their community.

“*Politicians determine what programs to fund, and health issues are not seen the same by every legislature*”

“*I think separating the science from the politics is the most important thing. There is no such thing as ‘alternate facts’ when it comes to science*”

“*The public sees politicians as wanting a win for their political party rather than focusing on the community’s health needs*”

#### 3.1.5. Transportation

Eleven participants identified long travel times, and several noted that especially in Kansas, patients must travel an hour or more to receive appropriate medical services. Two participants suggested clinics giving free ride passes for public transportation or even starting their affordable patient transportation services as solutions to transportation barriers (See Table 2). Along with providing means of vehicular transportation, many participants commented that it is essential for people to access safe sidewalks and biking lanes.

“*People without a vehicle or driver’s license are less likely to seek care due to lack of transportation*”

“*Having a safe area to walk or bike also promotes physical activity*”

“*Active forms of transportation can help close the gap between physical activity and transportation and make it safer, easier, and more convenient*“

#### 3.1.6. Poor Nutrition

Many participants discussed access to healthy and affordable food, and opportunities for proper nutrition. Thirteen participants identified poor nutrition as a barrier to public health and several suggested classes to teach children how to have a nutritious diet and properly prepare foods as a strategy to create healthy habits (See Table 2).

“*Also, food deserts are a huge problem. In rural areas a lot of people have to travel miles to get food. In [community name] most people have to go ten or more miles to find places to eat or groceries*”

“*Build infrastructure to offer more community gardens and farmers markets*”

#### 3.1.7. Expansion of Medicare and Medicaid

Six participants described how expanding Medicare and Medicaid in Kansas would impact public health. They stated that some people make just above the threshold to qualify for Medicare or Medicaid and cannot afford expensive health treatments. The overall consensus was that the expansion would help lift people out of poverty and provide them with healthcare.

“*I hope that Kansas will expand Medicaid, but it is unlikely because it is a politicized issue*”

### 3.2. Vulnerable Populations

#### 3.2.1. Poverty

Poverty was the most identified determinant of a vulnerable population in this study. Eighteen participants noted that people living in poverty are most likely to experience health disparities (See Table 3). Specific examples from participants were that low-income patients are less able to prioritize health in their budget, causing their problems to worsen until they become severe or even untreatable; if one cannot afford their medical services, they are also less likely to seek veterinary treatment for their pet; and that poor health can affect people of all income levels, but those in the low-income level are more likely to experience negative health outcomes due to lack of affordability.

“*They have a harder time gaining access to services, addressing issues, [and] getting appropriate services*”

#### 3.2.2. Elderly

Elderly people were the second most frequently identified vulnerable population in this study. Ten practitioners agreed that elderly populations present unique health challenges (See Table 3). Representation of and understanding the natural aging process and generational differences were specifically identified as important when serving the elderly. Participants who work with the elderly population suggested that ageism can impact patients’ access to providers, providers’ perceptions, and can influence how older adults seek healthcare.

“*Older people on fixed incomes find it challenging to afford routine checkups or live in a care facility*”

“*Older adults need transportation to and from appointments, and want to remain independent for as long as possible*”

#### 3.2.3. Marginalized and Minority Populations

Marginalized and minority populations were the third most frequently identified vulnerable population in this study. Thirteen participants described challenges faced by minority populations in their communities (See Table 3). Language barriers and cultural competency were important challenges identified and participants realized that communication must be approached with accuracy and engagement as core strategies. All participants agreed that minority populations are more likely to experience health inequities.

“*It is important to be culturally competent [in healthcare] to ensure equitable care*”

“*There is still very much a racial divide in the community*”

“*African American women have the highest rates of complications from pregnancy and the highest number of infant deaths, and it’s across America. This should be prioritized everywhere but particularly in areas with large populations of African American women*”

## 4. Discussion

Public health is local, and is provided and experienced at the local level. Our results demonstrate that local public health practitioners in Kansas are concerned with barriers to public health and the challenges faced by vulnerable people in their own communities. Overall, concerns with funding, equitable access to services and transportation, and both the politization and mistrust of public health were repeatedly identified by participants in this study. These concerns have not been extensively identified in public health in Kansas previously, but are recognized nationally both in urban and rural settings [5]. Many communities across the US face challenges of inequitable access to affordable healthcare, delaying care due to costs, and decreased opportunity or fair access to public health services [2,3,4,5,6,7].

In the US, disparities between rural and urban regions exist in public health services and health outcomes [4,5,6,7]. The County Health Rankings of rural and non-rural US counties has demonstrated differences in several indicators of health behaviors including level of exercise and diet, and the physical environment including access to healthy foods and recreational facilities [6]. Access to healthcare has also been identified as difficult in rural communities, including lack of services, financial burdens, greater poverty, and insufficient transportation [7]. Similar to burdens reported at the national level, social determinants of health for communities in Kansas are worse compared to the US as a whole, including poverty, household income, smoking adults, and lack of health insurance (see Table 1).

These concerns of barriers to public health and the ability to tackle health inequalities recognized by practitioners in this study have also been identified outside the US [38,39,40]. A study in Canada confirmed the challenges identified in the US and reported a weakening public health infrastructure damages the ability of public health to address health inequalities, particularly due to decreased funding of public health programs [41].

The participants in this study recognized vulnerable populations in their communities, and the importance of being culturally competent in order to eliminate health disparities. This correlates with public health challenges at the national level and indicates that Kansas public health experiences many of the same challenges and barriers identified in other parts of the US [38,39,40]. Furthermore, the results of this study reinforce the necessity that health equity is grounded in community-engaged and qualitatively informed work [42,43].

The qualitative thematic analysis employed in this study allowed researchers to understand phenomena and perceptions from the perspective of participants and use methodology to systematically analyze data and identify emerging patterns or themes [44,45]. There are limitations to this study. Qualitative studies can be subjective compared to quantitative studies, leading to error and bias [46,47,48]. First, researchers bring with them their unconscious biases and previous knowledge to the process of qualitative research, and therefore, they must be careful in all project stages, from interviewing to development of themes, to not influence the outcomes of the thematic analysis. Second, the results of this qualitative study are contextual and limited to Kansas and are defined by these geographical boundaries. Future studies to include more public health practitioners outside Kansas would enable other public health practitioners to determine if findings from this study could be transferred to investigate the needs of their own communities in other rural states.

## 5. Conclusions

Local public health practitioners serve as important resources for the analyses of public health practice and should be included in future studies to inform practice, not only at the local level, but also at state and national levels. Ultimately, our results indicate that there are significant barriers to public health practice in Kansas. This qualitative study furthers the evidence of both the knowledge and barriers that practitioners face in their communities in Kansas. Interviewing public health practitioners in Kansas provided an insight into the barriers they face and the vulnerable populations they serve. Incorporating tailored, community-based approaches and considering local public health barriers can help public health practitioners better serve their community.

## Figures and Tables

**Table 1:** Selected Social Determinants of Health in Rural and Urban Kansas.

Determinant	Rural KS	Urban KS	KS	USA *
Poverty Rate ^1^	12.9%	10.6%	12%	10.5%
Household Income ^2^	USD 65,100	USD 85,200	USD 77,400	USD 79,900
Adults Smoking ^3^	27.5%	17.4%	16.2%	20.8%
Exercise Opportunity ^4^	59.5%	80.1%	80.4%	52%
No Health Insurance ^5^	10.6%	9.8%	9.2%	9.2%

^1^ The percentage of people living below the federal poverty level [10,13,14]. ^2^ The estimated median family income [13,14]. ^3^ The percentage of adults who smoke [15,16]. ^4^ The percentage of individuals who live reasonably close to a park or recreational facility [17,18]. ^5^ The percentage of people without health insurance [19,20,21]. * United States (USA).

**Table 2:** Barriers of Public Health Identified by Kansas Public Health Practitioners.

Theme	Key Findings
Funding	Funding is necessary for health policies
	External variables for healthcare are expensive (transportation, insurance)
Education	Community not aware of their own health risks
	Community not aware of health programs
Accessibility and Affordability	Health insurance is not affordable for low-income citizens
	Access to affordable healthcare determines how likely people are to seek care
	Equity in health care access
Politics	Politicized health issues have caused distrust in public health officials
	Politicians determine funding
Transportation	People in need of transportation to access health resources
	Emphasis should be placed on walking and biking
Poor Nutrition	People need access to healthy and affordable food
Expansion of Medicare and Medicaid	Expanding Medicare and Medicaid will help low-income people to afford insurance

**Table 3:** Vulnerable Populations and Challenges Identified by Kansas Public Health Practitioners.

Population of Concern	Unique Challenges
People living in Poverty	Low-income adults are more likely to experience health disparities
Elderly People	Elderly citizens present unique health challenges Elderly in need of representation in health programs Elderly are susceptible to ageism in health care
Marginalized and Minority Populations	Unable to access appropriate services Physicians learning cultural competency Helping patients with a language barrier

## Data Availability

Data supporting the reported results can be requested from E.R.M.
